# Divergent roles of the regulatory subunits of class IA PI3K

**DOI:** 10.3389/fendo.2023.1152579

**Published:** 2024-01-22

**Authors:** Cho-Won Kim, Junsik M. Lee, Sang Won Park

**Affiliations:** ^1^ Division of Endocrinology, Boston Children’s Hospital, Boston, MA, United States; ^2^ Department of Pediatrics, Harvard Medical School, Boston, MA, United States

**Keywords:** PI3K, p85, glucose metabolism, insulin signaling, cancer

## Abstract

The regulatory subunit of phosphatidylinositol 3-kinase (PI3K), known as p85, is a critical component in the insulin signaling pathway. Extensive research has shed light on the diverse roles played by the two isoforms of p85, namely p85α and p85β. The gene *pik3r1* encodes p85α and its variants, p55α and p50α, while *pik3r2* encodes p85β. These isoforms exhibit various activities depending on tissue types, nutrient availability, and cellular stoichiometry. Whole-body or liver-specific deletion of *pik3r1* have shown to display increased insulin sensitivity and improved glucose homeostasis; however, skeletal muscle-specific deletion of p85α does not exhibit any significant effects on glucose homeostasis. On the other hand, whole-body deletion of *pik3r2* shows improved insulin sensitivity with no significant impact on glucose tolerance. Meanwhile, liver-specific double knockout of *pik3r1* and *pik3r2* leads to reduced insulin sensitivity and glucose tolerance. In the context of obesity, upregulation of hepatic p85α or p85β has been shown to improve glucose homeostasis. However, hepatic overexpression of p85α in the absence of p50α and p55α results in increased insulin resistance in obese mice. p85α and p85β have distinctive roles in cancer development. p85α acts as a tumor suppressor, but p85β promotes tumor progression. In the immune system, p85α facilitates B cell development, while p85β regulates T cell differentiation and maturation. This review provides a comprehensive overview of the distinct functions attributed to p85α and p85β, highlighting their significance in various physiological processes, including insulin signaling, cancer development, and immune system regulation.

## 1 Introduction

The insulin signaling pathway exhibits a high degree of conservation across different species, spanning from *C. elegans* to mammals. For example, insulin receptor (IR) and insulin growth factor 1 receptor (IGF1R) in mammals and abnormal dauer formation 2 (DAF2) in *C. elegans* respond to external signals and recruit insulin receptor substrate (IRS) or IRS-like adaptor (IST1), which then activate the PI3K-Akt-FoxO (PI3K: phosphatidylinositol 3-kinase, FoxO: forkhead family of transcription factor) axis in mammals or the AGE1/AAP1-Akt-DAF16 (AGE1: aging alteration 1, AAP1: AGE1 adaptor protein, DAF16: abnormal dauer formation 16) axis in *C. elegans* (1). In *D. melanogaster*, the PI3K signaling is regulated by the IR-Chico-Dp110/p60-Akt-FoxO pathway (1). The involvement of PI3K in chemotactic activities further exemplifies its evolutionary conservation. For instance, in *D. discoideum*, PI3K responds to chemoattractants and phosphorylates phosphatidylinositol (4,5)-bisphosphate (PI(4,5)P_2_) to phosphatidylinositol (3,4,5)-trisphosphate (PI(3,4,5)P_3_), which subsequently leads to actin polymerization and pseudopodium formation (2–4). A similar process occurs in mammals, where PI3Kγ in neutrophils responds to chemokines and chemotactic peptides, resulting in the generation of PI(3,4,5)P_3_, which in turn affects cell motility (5). These highlight the conservation and functional significance of the PI3K signaling pathway in different organisms and cellular processes.

Mammals exhibit a diverse range of isoforms for each subunit of PI3K, whereas organisms like *C. elegans* and *D. melanogaster* have only one form of PI3K (1). However, the precise roles and distinct functionalities associated with each isoform in the regulation of cellular processes remain to be fully understood. It is postulated that the presence of multiple isoforms in mammals has evolved as an adaptation to enable a broader range of control over the PI3K pathway, in order to accommodate the complexity of nutrient sensing and metabolism regulation in multicellular organisms (1). The specific functions and regulatory mechanisms of the different PI3K isoforms in mammals represent an area of ongoing scientific exploration.

PI3K activates its substrates by phosphorylating the 3-hydroxyl group of the inositol ring (6). PI3K can be classified into three groups based on their molecular structure and function (7, 8). **Table 1** provides a summary of the PI3K classification. Class I PI3K comprises a regulatory subunit and a catalytic subunit. Within class I PI3K, there are two sub-groups: class IA and class IB. The regulatory subunits of class IA PI3K consist of five variants: p85α, p55α, p50α, p85β, and p55γ. The presence of these variants adds complexity to the mammalian system, making it more intricate to identify their precise roles. 

**Table 1:** Classification of the PI3K family.

PI3K class	Regulatory subunit	Catalytic subunit	Catalyzed reaction
Class IA	p85α, p55α, p50α, p85β, and p55γ	p110α, p110β, and p110δ	• PI → PI(3)P • PI(4)P → PI(3,4)P_2_ • PI(4,5)P_2_ → PI(3,4,5)P_3_
Class IB	p101 and p84 (p87PIKAP)	p110γ	• PI → PI(3)P • PI(4)P → PI(3,4)P_2_ • PI(4,5)P_2_ → PI(3,4,5)P_3_
Class II	None	PI3K-C2α, PI3K-C2β, and PI3K-C2γ	• PI → PI(3)P • PI(4)P → PI(3,4)P_2_
Class III	Vps15	Vps34	• PI → PI(3)P

**Figure 1:**
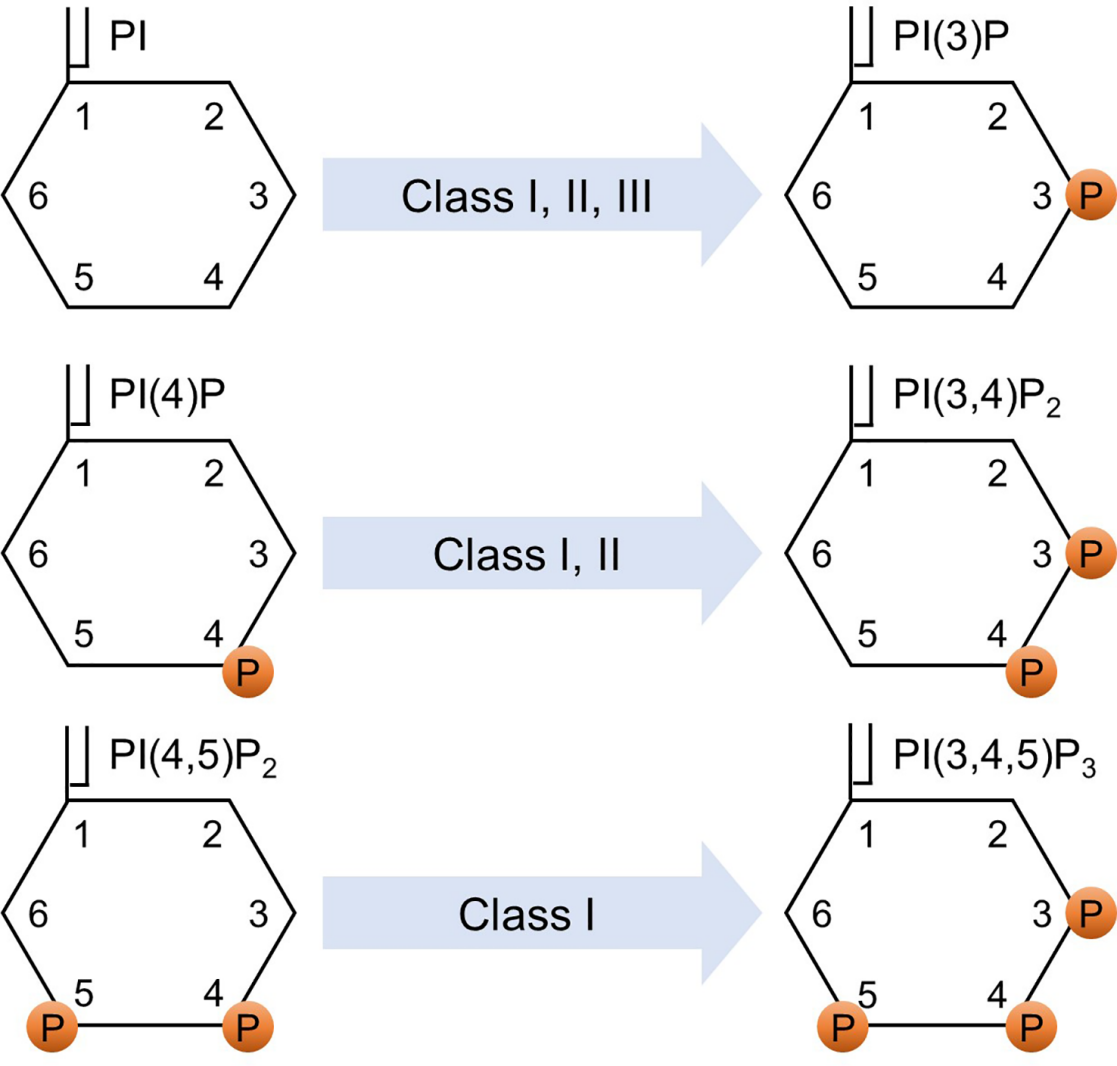
Phosphorylation of phosphoinositides. Various classes of PI3K phosphorylate various phosphoinositides. PI, phosphatidylinositol; PI(4)P, phosphatidylinositol-4-phosphate; PI(4,5)P_2_, phosphatidylinositol 4,5-bisphosphate; PI(3)P, phosphatidylinositol 3-phosphate; PI(3,4)P_2_, phosphatidylinositol 3,4-bisphosphate; PI(3,4,5)P_3_, phosphatidylinositol 3,4,5-trisphosphate; and P indicates a phosphorylated site.

p85α and its splicing variants, p55α and p50α, are encoded by *pik3r1*, p85β is encoded by *pik3r2*, and p55γ is encoded by *pik3r3*. p85α, p55α, and p85β are expressed ubiquitously, while the expression of p50α and p55γ is restricted to specific tissues, such as the liver, kidney, brain, and testis (9–11). The catalytic subunit of class IA PI3K comprises three variants: p110α, p110β, and p110δ. Class IB PI3K consists of the regulatory subunits of p101 or p84 (also known as p87PIKAP) and the catalytic subunit of p110γ (1, 12, 13). Class I PI3K phosphorylates phosphatidylinositol (PI), phosphatidylinositol-4-phosphate (PI(4)P), and phosphatidylinositol 4,5-bisphosphate (PI(4,5)P_2_) to generate phosphatidylinositol 3-phosphate (PI(3)P), phosphatidylinositol 3,4-bisphosphate (PI(3,4)P_2_), and phosphatidylinositol 3,4,5-trisphosphate (PI(3,4,5)P_3_), respectively (1, 14). Determining the affinity is a challenging task due to the relatively low binding specificity and affinity of the PI-binding domains toward different PIs, coupled with their tendency to interact with other protein ligands (15). While further investigations are needed to fully validate the precise affinities for different PIP molecules, based on the Km values, which represent the association between the reaction rate and the substrate concentration, it was observed that PI(4,5)P_2_ has the lowest Km compared to PI(4)P and PI (16). This indicates that the affinity of the enzyme for its substrate is higher for PI(4,5)P_2_ compared to PI(4)P and PI. The preferential production of PI(3,4,5)P_3_ from PI(4,5)P_2_ is well-documented and has been proven to be a critical catalytic reaction in class I PI3K signaling (1, 17–19). Class II PI3K has three catalytic isoforms: PI3K-C2α, PI3K-C2β, and PI3K-C2γ, and does not have a regulatory subunit (20). Class II PI3K phosphorylates PI and PI(4)P to produce PI(3)P and PI(3,4)P_2_, respectively (1, 21). Class III PI3K consists of a catalytic subunit, known as vacuolar protein sorting 34 (Vps34) (22). Vps15 functions as a regulator of Vps34 activity. However, it differs from other regulatory subunits in that it itself acts as a kinase (23, 24). Class III PI3K phosphorylates PI to generate PI(3)P(1). **Figure 1** shows the phosphorylation of different classes of phosphoinositides by PI3K.

Since its initial discovery in 1984 (25), extensive research has been conducted to elucidate the diverse roles of PI3K in intracellular signaling pathways, including insulin signaling, proliferation, differentiation, survival, and apoptosis (1, 6, 26–28). In 1991, the p85 regulatory subunit was identified as a protein that regulates the interaction between PI3K and platelet-derived growth factor β (PDGFβ) receptors (29). The primary function of the p85 regulatory subunit is to recruit PI3K to the plasma membrane (30). It physically binds to the p110 catalytic subunit and stabilizes it by maintaining it in a low activity state (31). The p85 subunit interacts with p110 through its N-terminal SH2 (nSH2), inter Src homology 2 (iSH2), and C-terminal SH2 (cSH2) domains (32–34). The SH2 domain of p85 recognizes and binds to phosphorylated YXXM motifs found in tyrosine kinases, such as IRS proteins (35). Phosphorylation of the tyrosine 688 residue on p85 induces a conformational change in the p85-p110 complex, relieving the inhibitory effect of p85 on p110. Consequently, this activation of p85 leads to the increased catalytic activity of PI3K (31, 34, 36, 37).

The study of feedback mechanisms in the PI3K pathway is paramount due to their significant role in maintaining the delicate balance of PI3K signaling and their potential impact on various cellular processes. Disruptions in these feedback mechanisms have been implicated in the development of pathological conditions, such as cancer, which highlights the need for further investigation of these feedback mechanisms. Aberrant activation of PI3K leads to sustained activation of Akt. In such event, Akt phosphorylates tuberous sclerosis protein 2 (TSC2). The inhibition of TSC2 results in the activation of mammalian target of rapamycin complex 1 (mTORC1), which then activates S6 kinase (S6K). S6K, in turn, phosphorylates IRS1 at its serine residue, inhibiting its activity. This subsequently reduces PI3K activity (1). This intricate feedback mechanism, where the IRS-PI3K pathway is inhibited by the activation of mTOR-raptor, serves as a crucial brake to prevent uncontrolled cellular transformation and halt the vicious cycle. There are several additional examples of negative feedback loops, in which the IRS-PI3K pathway is inhibited. The activation of c-Jun N-terminal kinase (JNK) activates mTOR and induces serine phosphorylation of IRS1, which leads to the attenuation of Akt activity (1, 38, 39). Another feedback mechanism can be explained by the role of growth factor receptor-bound protein 10 (Grb10) (40). Grb10 is known to play a role as a negative regulator of insulin signaling and its absence leads to hyperactivation of the PI3K-Akt pathway in skeletal muscle and adipose tissue (41). It has been shown that mTORC1 mediates the phosphorylation and accumulation of Grb10, which leads to reduced IRS tyrosine phosphorylation, and thereby reduces PI3K recruitment (40). Also, in the case of amino acid deprivation, inhibitor of nuclear factor κ-B kinase (IKK) phosphorylates p85α at the serine 690 residue, which blocks the binding of p85 to IRS proteins, consequently reducing PI3K activity (42). Akt itself is also involved in the negative feedback regulation of PI3K. It has been shown that Akt directly phosphorylates IRS2 on serine 306 and 577 residues in 3T3-L1 adipocytes, which limits the interaction of IRS and IR, resulting in decreased PI3K activity and reduced production of PI(3,4,5)P_3_ (43). Akt signaling activates mTORC1 and S6K, which then suppresses IRS1 expression, serving as an additional regulatory mechanism (44). Once FoxO1 is phosphorylated by Akt, FoxO1 cannot further upregulate receptor kinases that are activated by PI3K, such as IR and HER3 (45, 46). Additionally, a recent study revealed that physiological or oncogenic activation of the PI3K signaling pathway utilizes mTOR/eukaryotic translation initiation factor 4E-binding protein 1 (4E-BP1) to increase the expression of phosphatase and tensin homolog (PTEN) (47), which dephosphorylates PI(3,4,5)P_3_ to PI(4,5)P_2_, thereby reducing the activity of 3-phosphoinositide-dependent protein kinase-1 (PDK1) and Akt (43, 48, 49). Of interest, sustained inhibition of PI3K-Akt signaling leads to a resurgence of Akt activity, indicated by increased phosphorylation of Akt at serine 473 and at threonine 308 residues in a breast cancer cell line (47). Therefore, although the feedback mechanism involving PTEN serves to restrict the duration and impact of the PI3K pathway, in the context of tumor treatment that utilizes PI3K inhibitors, this regulatory mechanism can lead to decreased PTEN activity, ultimately reducing the effectiveness of prolonged treatment. 

Positive feedback mechanisms are involved in amplifying or sustaining the initial activation of PI3K. In endothelial cells, Akt activation stimulates endothelial nitric oxide synthase (eNOS), resulting in increased production of nitric oxide (NO) (50). NO, in turn, upregulates vascular endothelial growth factor (VEGF) signaling, which further activates the PI3K-Akt pathway (51). This positive feedback loop is utilized by endothelial cells to promote cell proliferation. Inhibiting the synthesis of NO resulted in a decrease in proliferation specifically induced by VEGF in human umbilical vein endothelial cells (52). Another example has been reported in head and neck squamous cell carcinoma. PI3K-Akt signaling downstream of the epidermal growth factor receptor (EGFR) activates mTORC1, which subsequently activates the IKK and nuclear factor-κB (NF-κB). Activation of mTORC1/NF-κB, in turn, increases the expression of EGFR, leading to sustained proliferation of cancerous cells (53). Additionally, a positive feedback mechanism was reported with the role of WIPI2 (WD repeat domain, phosphoinositide-interacting protein 2), a protein involved in the process of autophagy. PI3K and WIPI2 promote the recruitment of each other, the loop of which facilitates the lipidation of LC3, a protein that is involved in cargo sequestration and autophagosome formation (54). 

**Figure 2:**
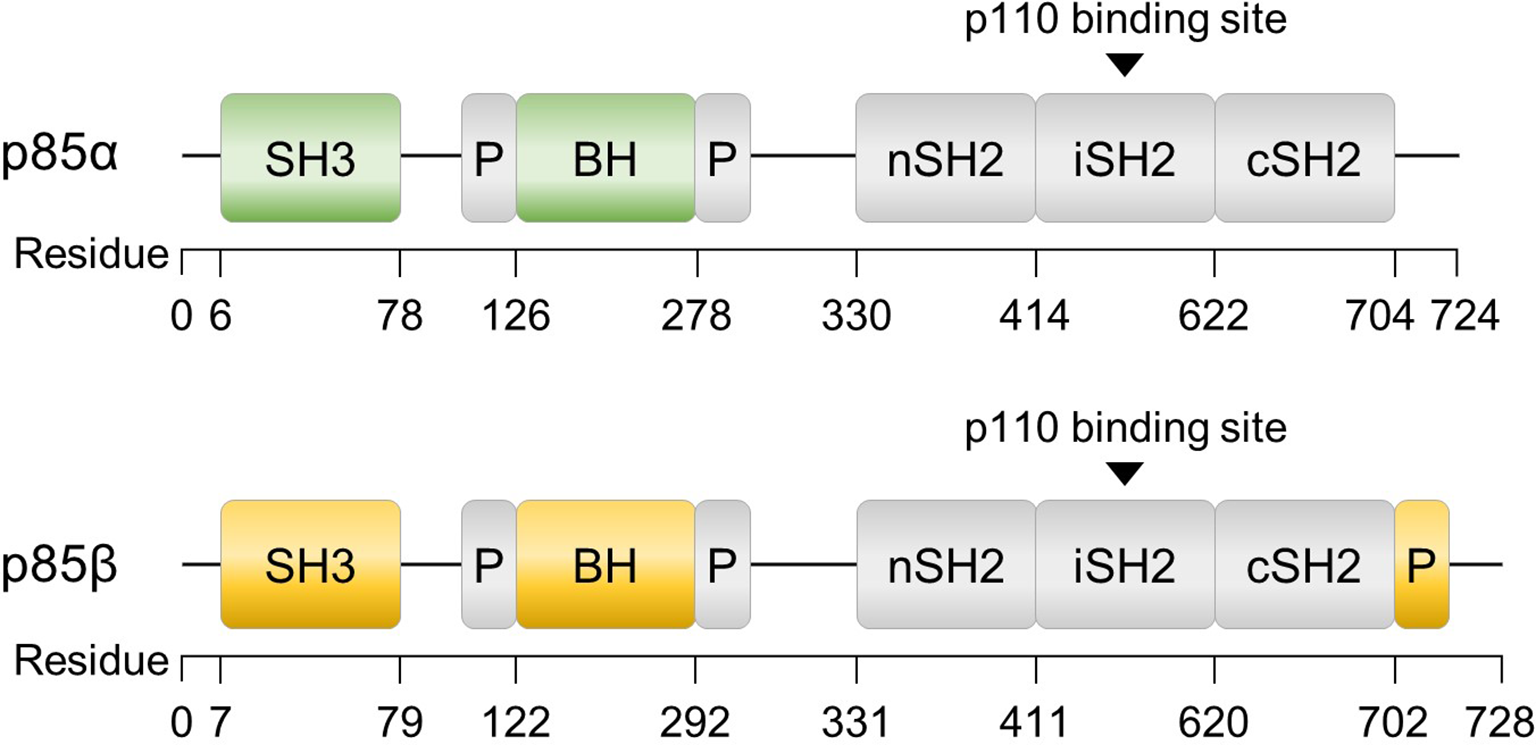
Structures of p85α and p85β, the regulatory subunits of PI3K. The domains consist of SH3 (Src homology 3), BH (B cell receptor homology) flanked by a proline-rich region (P), nSH2 (N-terminal SH2), iSH2 (inter-SH2), and cSH2 (C-terminal SH2). Additionally, p85β contains a proline-rich region at the C-terminus.

In addition to multiple regulatory mechanisms governing PI3K activity, the p85 regulatory subunits, p85α and p85β, also exhibit unique functions within distinct signaling pathways and biological processes, including insulin signaling, cancer progression, and lymphocyte development (55–58). Although p85α and p85β share similar structural motifs (**Figure 2**), there are notable differences between the two isoforms. The SH3 and B cell receptor homology (BH) domains of p85α and p85β exhibit only 37% homology, and p85β possesses a proline-rich region in its C-terminal region. While the precise function of each domain requires further investigation, these structural and sequential disparities may contribute to the growing body of evidence suggesting functional distinctions between the two isoforms (59). Understanding the roles of these subunits is essential for comprehending the intricate regulation and signaling outcomes associated with PI3K activity. This review discusses the diverse roles of p85α and p85β.

## 2 Divergent roles of p85α and p85β in insulin signaling

Extensive research has been conducted over the past two decades to study the role of p85 in metabolism and insulin signaling, frequently employing genetically modified animal models. Studies using mice lacking the *pik3r1* gene exhibit perinatal mortality accompanied by hepatocytes and brown adipose tissue (BAT) necrosis, chylous ascites, as well as muscle tissue abnormalities, such as enlarged skeletal fibers and cardiac muscle calcification (57, 60). However, under pathogen-free conditions, these mice manage to survive and demonstrate increased insulin sensitivity, hypoglycemia, reduced fasting insulin levels, normal body weight, and unaltered fat mass (60–63). Furthermore, they exhibit enhanced glucose transport in skeletal muscle and adipocytes attributed to the augmented translocation of glucose transporter type 4 (GLUT4) (61). Heterozygous whole-body deletion of *pik3r1* promotes insulin signaling (64). Reduced *pik3r1* is sufficient to ameliorate high-fat diet-induced insulin resistance, enhance insulin signaling in white adipose tissues and skeletal muscle, and also improve whole-body insulin sensitivity (65).

Liver-specific *pik3r1* knockout mice display improved insulin sensitivity and glucose tolerance with decreased levels of serum-free fatty acids and triglycerides (66). These mice exhibit reduced PTEN activity and increased Akt activation in response to insulin, regardless of diet composition, despite decreased IRS1, IRS2, and PI3K activities (66, 67). BAT-specific *pik3r1* knockout mice on a high-fat diet display improved insulin sensitivity but no changes in glucose tolerance (68). These mice demonstrate enhanced thermogenic function, increased browning of inguinal white adipose tissue (iWAT), reduced body weight and fat content, lower glucose and insulin levels, and decreased liver steatosis. They exhibit increased mRNA and protein levels of IRβ and decreased JNK phosphorylation in response to insulin stimulation (68). Overall, these findings suggest that alterations in hepatic and adipose signaling pathways affect the insulin sensitivity levels in mice with homozygous and heterozygous deletion of *pik3r1*. 

Five regulatory subunits, namely p85α, p50α, p55α, p85β, and p55γ, interact with receptor tyrosine kinases, such as IR, PDGF, and EGFR (69, 70), with varying binding affinities. Among the three p85α variants, p50α exhibits the highest PI3K activity (61, 70, 71). Conversely, p55γ displays the least interaction with IRS1 in response to insulin (69). Deletion of p85α in mice, excluding its splicing variants, results in diverse phenotypes depending on the specific tissues involved. Mice with a whole-body deletion of p85α exhibit enhanced insulin sensitivity in muscles, but not in the liver. This differential response to insulin can be attributed to the decreased expression levels of p50α in muscles because p50α and p55α can compensate for the deficiency of p85α by binding to IRS1/2 and activating PI3K signaling (61, 72). p85β and p55γ do not fulfill a compensatory role in the absence of p85α (61). Despite no alterations in PI3K activity in p85α knockout mice, they exhibit increased hepatic gluconeogenesis (72).

Mice with a deletion limited to the splice variants of *pik3r1*, specifically p50α and p55α, display enhanced insulin sensitivity but no significant difference in glucose tolerance (73). In the fed state, there are no notable changes in glucose and insulin levels, while insulin levels are decreased in the fasting state. The absence of p50α/p55α leads to increased glucose uptake in the extensor digitorum longus muscle and adipocytes in response to insulin. Despite comparable body weights to wild-type mice, these knockout mice display decreased adiposity (73). Additionally, when p50α/p55α knockout mice are treated with the hypothalamic toxin gold thioglucose (GTG), which typically induces hyperphagia, obesity, and insulin resistance (74, 75), they demonstrate decreased insulin levels and epididymal fat, compared to GTG-treated wild-type control mice (73). Conversely, overexpression of p55α in skeletal muscle in mice does not impact body weight, blood glucose levels, whole-body glucose tolerance, or skeletal muscle insulin sensitivity (76). Insulin-stimulated Akt phosphorylation levels remain normal in skeletal muscle and liver of these mice (76). Exploring the effects of p50α deletion alone would be of particular interest. Further investigations are required to gain a better understanding of the functions of each isoform and their responses to specific biological conditions, but studies suggest that each isoform plays various roles in an organ-specific manner.

Heterozygous deletion of whole-body *pik3r1* improves diabetic symptoms caused by heterozygous disruption of IR and IRS1 genes (64). Conversely, reintroducing p85α in liver-specific *pik3r1* knockout mice leads to elevated fasting blood glucose and insulin levels, decreased glucose tolerance, and reduced phosphorylation of Akt (67). However, skeletal muscle-specific deletion of p85α does not improve insulin sensitivity, and they display normal body weight, insulin and glucose levels, and fat content (77). Although Akt phosphorylation is not significantly reduced in this knockout model, there is a slight decrease in glycogen synthase kinase 3β (GSK3β) phosphorylation in response to insulin, albeit with some variations in the results (77). In an obese condition, it gives a different outcome. The upregulation of p85α in the liver of obese mice results in decreased blood glucose levels and improved glucose homeostasis (78).

Mice with a deletion of the *pik3r2* gene display improved insulin sensitivity and a moderate decrease in blood glucose and insulin levels, with no differences in glucose tolerance levels (79). Of interest, deletion of the *pik3r2* gene does not affect glucose homeostasis when mice are fed on a high-fat diet (65). On the other hand, the upregulation of p85β in the liver of obese mice leads to improved glucose homeostasis (78). In conclusion, whole-body deletion of *pik3r2* in normal chow diet-fed mice and upregulation of hepatic p85β in obese mice lead to improved glucose homeostasis.

Deletion of hepatic *pik3r1* and whole-body *pik3r2* results in reduced insulin sensitivity and glucose tolerance (80). These mice exhibit hyperglycemia and hyperinsulinemia in both fasted and fed states. Impaired Akt activation upon insulin stimulation leads to reduced phosphorylation of FoxO1 and GSK3β, along with increased expression of hepatic gluconeogenesis genes, including phosphoenolpyruvate carboxykinase 1, glucose-6-phosphatase, and fructose 1,6-bisphosphatase 1 (80). However, while the deletion of p85α in skeletal muscle does not affect glucose homeostasis, further deleting p85β in whole-body leads to impaired glucose tolerance in both normal chow and high-fat diet fed mice (77). These mice maintain normal blood glucose and insulin levels, systemic insulin sensitivity, and body weights. However, they exhibit impaired insulin sensitivity in muscle, characterized by reduced Akt activation and decreased GSK3α/β phosphorylation in response to insulin or IGF1 stimulation. They display serum hyperlipidemia and impaired muscle growth, as evidenced by reduced fiber size and muscle weight (77). Targeted deletion of p85α/β exclusively in muscle tissues may provide valuable insights and more information on the specific roles of p85α and p85β.

The differential response of p85α and p85β to insulin stimulation has also been revealed through the involvement of a protein called bromodomain-containing protein 7 (BRD7). It has been demonstrated that BRD7, initially recognized as a tumor suppressor (81), enhances the phosphorylation of Akt (82). However, it exerts different effects depending on which isoform is present. In mice with deletion of p85β, overexpression of BRD7 in the liver during high-fat diet challenge does not affect the phosphorylation of Akt at the basal state without any stimulation. However, in mice with a lack of hepatic p85α, the upregulation of BRD7 leads to increased Akt phosphorylation under the same conditions (83). On the other hand, the upregulation of BRD7 leads to an increase in Akt phosphorylation upon insulin stimulation in the liver of high-fat diet-induced obese p85β knockout mice (83). However, in high-fat diet fed hepatic p85α knockout mice, the effect of BRD7 on Akt phosphorylation in response to insulin stimulation is abolished (83). These observations indicate that p85α plays a role in mediating the effect of BRD7 on Akt phosphorylation in response to insulin, while p85β is involved in the regulation during the basal state without responding to external stimuli. Additionally, immunoprecipitation of PC12 cell lysates using antibodies specific to each of the five regulatory subunits revealed that p85α, p55α, and p50α exhibited increased PI3K activity upon insulin stimulation, whereas p85β or p55γ did not show the same response (70). These findings further support the notion that p85α is primarily involved in insulin response, while p85β influences the basal levels without the stimulation. PI3K has emerged as a highly promising therapeutic target in various diseases, such as diabetes and cancer. However, due to variations in their effects depending on tissue types, nutrient availability, and splice variants, further validations are needed. p85 is also known to be a player in the transcriptional regulation of numerous genes involved in glucose metabolism (72, 80, 84), reflecting its complex nature. This highlights the need for in-depth studies to elucidate the specific functions of these genes. In **Table 2**, we provide a summary of the metabolic phenotypes of p85 manipulation observed in various animal models.

**Table 2:** Metabolic phenotypes of p85 knockouts or transgenic mice.

Isoform	Tissue	Manipulation	Background	Diet	Metabolic phenotypes	Ref.
*Pik3r1*	Whole-body	Knockout	Mixed background (C57BL/6 and CBA)	NCD	Increased insulin sensitivity in muscle and adipose tissues.	(61)
*Pik3r1*	Whole-body	Knockout	Mixed background (C57BL/6J and CBA)	HFD	Improved glucose homeostasis. Decreased fasting insulin levels.	(63)
*Pik3r1*	Whole-body	Heterozygous knockout	Mixed background (C57BL/6J and CBA; or 129Sv and C57BL/6)	NCD	Increased glucose production. Improved insulin signaling.	(64, 72)
*Pik3r1*	Whole-body	Heterozygous knockout	C57BL/6SVJ	HFD	Improved insulin resistance.	(65)
*Pik3r1*	Liver	Knockout	Mixed background (129Sv, C57BL/6 and FVB)	NCD	Improved glucose tolerance and insulin sensitivity. Decreased serum free fatty acids and triglycerides levels.	(66)
*Pik3r1*	Liver	Knockout	Mixed background (129Sv, C57BL/6 and FVB)	HFD	Improved glucose tolerance.	(67)
*Pik3r1*	Brown adipose tissue	Knockout	C57BL/6	HFD	No difference in glucose tolerance level. Improved insulin sensitivity. Improved thermogenic functionality. Decreased body weight and fat content. Decreased blood glucose and insulin levels. Reduced liver steatosis.	(68)
p85α	Muscle	Knockout	Mixed background (129Sv, C57BL/6, and FVB)	NCD	No improvement in insulin sensitivity No effect on body weights, insulin, and glucose levels. No difference in body fat content, fasting serum free fat acids level, and fasting serum triglycerides level.	(77)
p85α	Liver	Overexpression	B6.Cg-Lep^ob^/J (*Ob*/*Ob*)	NCD	Decreased blood glucose levels. Improved glucose tolerance and insulin sensitivity.	(78)
*Pik3r2*	Whole-body	Knockout	Unspecified	NCD	Improved insulin sensitivity. Moderately decreased blood glucose and insulin levels. No effect on glucose tolerance.	(79)
*Pik3r2*	Whole-body	Knockout	Mixed background (C57BL/6J and CBA)	HFD	No improvement in glucose homeostasis.	(65)
p85β	Liver	Overexpression	B6.Cg-Lep^ob^/J (*Ob*/*Ob*)	NCD	Decreased blood glucose levels. Improved glucose tolerance and insulin sensitivity.	(78)
*Pik3r1*/ *Pik3r2*	Liver	Double knockout	Mixed background (129Sv, C57BL/6, and FVB)	NCD	Reduced glucose tolerance and insulin sensitivity. Hyperglycemia and hyperinsulinemia at both fasting and fed states.	(80)
*Pik3r1*/ *Pik3r2*	Muscle *(Pik3r1*) and Whole-body *(Pik3r2*)	Double knockout	Mixed background (129Sv, C57BL/6, and FVB)	HFD	Impaired glucose tolerance and muscle insulin sensitivity.	(77)

HFD, High-fat diet; NCD, Normal chow diet.

## 3 The regulatory roles of p85 in the PI3K pathway

p85 exists in monomers (p85α or p85β), homodimers (p85α-p85α or p85β-p85β), heterodimers (p85α-p85β), or in complexes with other proteins (e.g., p85α-p110). These different forms of p85 contribute to the regulation of the stability and activity of p85 in the PI3K signaling. For instance, an excessive accumulation of monomeric p85 may exacerbate the inhibition of PI3K activity. Of note, monomeric p85s are unstable. The stability of p85 monomers is enhanced through dimerization (85). This instability of p85 monomers may contribute to optimizing the overall functionality of the PI3K pathway. By selectively reducing free p85, cells can maintain a balanced ratio of p85 to p110, thereby enhancing the PI3K pathway. In other words, it is possible that cells employed a compensatory mechanism of preferentially decreasing the levels of free p85 compared to p85 bound to p110 to counteract the potential hindrance caused by excessive p85 monomers and prevent further impairment in situations where PI3K is diminished in the system (85).

The homodimerization of p85 is facilitated by interaction through the SH3 domain-proline rich motif (PR1) or BH-BH domains (86, 87). Unlike p85-p110 complex, p85 homodimers can negatively regulate the PI3K pathway by competing with the E3 ligase WW domain-containing protein 2 (WWP2) for binding to PTEN. The binding of p85 homodimers to PTEN leads to reduced WWP2-mediated proteasomal degradation of PTEN (86). Consequent increased stability of PTEN enhances the rate of dephosphorylation of PI(3,4,5)P_3_, resulting in reduced Akt phosphorylation at serine 473 (86). This demonstrates the effect of different forms of p85 on PI3K signaling.

In addition to homodimerization, monomeric p85 can exert a negative regulation on the PI3K pathway by binding to small ubiquitin-related modifier (SUMO) 1 and SUMO 2 proteins through its iSH2 domains (88). Further analysis of the p85β sequence identified lysine residues at 535 and 592 positions as the key sites responsible for this interaction. Conjugation of p85 to SUMO proteins leads to a reduction in its phosphorylation at tyrosine 458 and 199 residues (37, 88), as well as downstream phosphorylation events involving Akt and FoxOs (88).

The activity of p85 can be further modulated by various stimulations and mechanisms, such as insulin. For example, p85 heterodimers are dissociated in response to insulin stimulation (78). This dissociation increases the availability of p85 to interact with other proteins, including p110, thereby facilitating increased PI3K activity upon insulin stimulation. Moreover, p85α can be phosphorylated by IR or non-receptor tyrosine kinase, such as activated cell division control protein 42 (CDC42)-associated kinase (ACK), at their tyrosine 368, 580, and 607 residues (89) or at tyrosine 607 residue, respectively (90). This phosphorylation prevents p85 from undergoing ubiquitination, resulting in increased PI3K activity (90). Taken together, the balance and interaction between different forms of p85, along with post-translational modifications and external stimuli, intricately regulate the activity of PI3K and its downstream signaling pathways.

## 4 The significance of the ratio of p85 to p110 in PI3K signaling

The stoichiometric balance between the levels of p85 and p110 introduces an additional layer of complexity to the dynamics and outcomes associated with p85. This balance refers to the relative quantities of p85 and p110 within cells. Different tissues exhibit variations in the levels of p85 isoforms relative to p110. In many cells, the abundance of p85s exceeds that of p110 (64, 91). Specifically, p85α is more abundant than p110 in the liver and mouse embryonic fibroblasts, while p85β is more abundant than p110 in the brain, the lung, bone marrow, the liver, fat, skeletal muscles, and mouse embryonic fibroblasts (MEFs) (64, 92). This imbalance leads to a competition between excess p85 monomers and the p85–p110 complex for binding to activated IRS1 (56, 93). The binding of p85 monomers to IRS proteins inhibits the interaction between p85-p110 and IRS, resulting in reduced PI3K activity (64). The disruption of the PI3K-IRS complex by p85 provides an explanation for the observed improvement in insulin sensitivity upon deleting *pik3r1* in the liver (66).

While the binding partners of p85 and the ratio of p85 to p110 play crucial roles in the regulation of PI3K, reducing the amount of p85 does not alter the quantity of the p85-p110 complex as long as the amount of p85 remains higher than that of p110 (91). This is due to the irreversible nature of the binding between p85 and p110 (16, 85), as demonstrated in MEFs isolated from *pik3r1* heterozygous knockout mice. Both the wild-type and *pik3r1* heterozygous knockout cells displayed the same amount of PI3K, regardless of the p85 amounts (91). Therefore, the key difference lies in the availability of excess free p85. In wild-type cells, there is a larger pool of free p85 that can potentially block IRS sites, while in *pik3r1* heterozygous knockout cells, there is a lower abundance of excess free p85. Consequently, heterozygous cells may have a relatively higher portion of p85-p110 complexes available to bind to IRS compared to wild-type cells (91). Therefore, the regulation of PI3K by p85 involves factors, such as the ratio of p85 to p110 and the stability of free p85.

Recent discoveries have revealed the involvement of various p85 binding partners in the regulation of PI3K activity. For instance, the interaction between p85α/β and BRD7 has been identified as a significant contributor to this regulatory mechanism (82). BRD7 binds to p85 through the iSH2 domain and facilitates the transportation of p85 to the nucleus without affecting the nuclear translocation of p110 (82, 94). This suggests that BRD7 may decrease the cytoplasmic ratio of p85 to p110 by sequestering p85 in the nucleus (95). The outcome of this phenomenon can vary depending on the cell type and the relative abundance of p85 and p110. In the liver of obese mice, the upregulation of BRD7 promotes the interaction between BRD7 and p85, leading to increased nuclear translocation of p85. This balances the p85-p110 ratio, thereby improves PI3K signaling, resulting in enhanced Akt phosphorylation at threonine 308 and serine 473 residues (82, 83). Conversely, in the HeLa cervical cancer cell line, the sequestration of p85 in the nucleus by overexpression of BRD7 reduces PI3K activity, and leading to decreased Akt phosphorylation. Depletion of BRD7, on the other hand, increases PI3K signaling (94), possibly due to similar levels of p85 and p110 in this cell line. In summary, the balance between p85 and p110 levels plays a crucial role in the regulation of PI3K activity. Interactions with binding partners further modulate the subcellular localization and function of p85 isoforms in different cellular contexts.

## 5 The function of p85 independent of its role as a regulatory subunit of PI3K

Several reports have documented the roles of p85α and p85β beyond their functions as components of PI3K. One notable role is their involvement in the maintenance of endoplasmic reticulum (ER) homeostasis. p85α and p85β have been shown to interact with a transcription factor called the spliced form of X-box binding protein-1 (XBP1s), which serves as one of the master regulators for ER function (78). The interaction between p85 and XBP1s is crucial for the nuclear translocation and activity of XBP1s (78, 96). In the conditions of obesity and type 2 diabetes, upregulation of p85α and p85β in the liver alleviates ER stress by enhancing the nuclear translocation of XBP1s, which induces the transcription of ER chaperone genes involved in proper protein folding (78). Moreover, the liver-specific deletion of p85α impacts the activity of key components of the unfolded protein response (UPR) pathway, such as the inositol-requiring enzyme 1α (IRE1α) and activating transcription factor 6α (ATF6α) (96). Deletion of p85α reduces IRE1α phosphorylation and hampers ATF6α nuclear translocation (96), resulting in decreased endoribonuclease activity of IRE1α and reduced activity of ATF6α as a transcription factor. These observations collectively support the involvement of p85α in the regulation of ER homeostasis.

Furthermore, p85α has been implicated in the regulation of the stress kinase pathway during insulin resistance and stress conditions. This activation occurs independently of p85’s role in the PI3K complex under specific stimuli, such as insulin and tunicamycin. It was shown that the N terminus and SH2 domains of p85α are required for the activation of JNK by CDC42 and MKK4 (mitogen-activated protein kinase-kinase 4), highlighting the communication between the PI3K pathway and cellular stress responses (83).

Additionally, it was shown that p85 contributes to the stabilization of BRD7 (83). The expression levels of BRD7 were found to be low in the absence of p85 proteins in MEFs derived from p85α/β double knockout mice with a shorter half-life compared to wild-type mice. Co-expression of p85 and BRD7 led to a more stable and robust expression of BRD7 compared to when BRD7 was upregulated without p85 (83). These findings highlight the multifaceted functions of p85 beyond its classical role as a regulatory subunit of PI3K.

## 6 Distinct roles of p85α and p85β in cancers

In the context of cancer, p85α and p85β have demonstrated contradictory roles. The majority of studies suggest that p85α functions as a tumor suppressor. Decreased p85α levels have been detected in various human cancers, including prostate, lung, ovarian, bladder, breast, and liver cancers (97–100). The absence of p85α has been associated with increased tumor development in different tissues. For example, liver-specific p85α knockout mice have shown an elevated incidence of spontaneous hepatocellular carcinoma (HCC) with lung metastasis (97). Additionally, knockout of *pik3r1* in mice has been found to enhance tumor formation driven by the activation of human epidermal growth factor receptor 2 (HER2) (99). Disrupting the inhibitory effect of p85α on p110 by mutating the asparagine residue at the 564 position within the iSH2 domain has been shown to promote cell proliferation, survival, and Akt phosphorylation in lymphocytes (101). Additionally, it was shown that the level of p85α is downregulated in human bladder cancer cells (100). The overexpression of p85α led to suppression of cell invasion, while having no effect on cell migration. Conversely, the knockdown of p85α promoted invasion, suggesting that p85α acts as an inhibitor of invasion in the bladder cancer cells (100). The mechanism underlying invasion involves c-Jun inactivation by p85 knockdown, resulting in downregulation of miR-190 and subsequent degradation of ATG7 (the autophagy-related protein) mRNA, leading to reduced autophagy. This cascade of events leads to the upregulation of tissue inhibitor of metalloproteinase-2 (TIMP2), which acts to prevent breakdown of the extracellular matrix, and inactivation of matrix metalloproteinase-2 (MMP2), a zinc-dependent endopeptidase that acts to promote cancer progression by facilitating tumor to form metastases, and the regulation of TIMP2 and MMP2 contributes to the inhibition of bladder cancer invasion (100). These findings collectively support the role of p85α as a tumor suppressor in various cancer types.

On the contrary, p85β has been identified as an oncogene. Increased expression of p85β has been observed in several cancer types, including breast, endometrial, colon, ovarian, and lung cancers (10, 102–106). Overexpression of p85β in primary avian fibroblasts has been shown to significantly increase cell proliferation, with its oncogenic activity driven by the activation of PI3K and target of rapamycin (TOR) signaling pathways (104). In ovarian cancer cells, upregulation of p85β has been linked to increased proliferation, colony formation, and invasion, while depletion of p85β using small interfering RNA (siRNA) has been shown to reverse these effects (103). In colon and breast cancers, overexpression of p85β increased PI(3,4,5)P_3_ and phosphorylated Akt levels, which in turn enhanced cell invasion and accelerated the progression of tumors (10). Moreover, increased p85β expression in severe combined immunodeficient (SCID) mice through retroviral infection of bone marrow accelerated tumor progression in a thymic lymphoma model, resulting in earlier tumor onset, reduced lifespan, and a higher incidence of spleen metastases compared to control (10). Notably, increased nuclear translocation of p85β has been observed in colon, lung, and breast cancer cell lines, resulting in increased protein stability of the enhancer of zeste homolog (EZH), which is a known oncoprotein (107). Inhibition of p85β’s nuclear translocation through two point mutations on its lysine 477 and arginine 478 residues has been shown to suppress the proliferation of DLD1 colorectal cancer cells and impede tumor growth (107). These findings collectively suggest that p85β functions as an oncogene and may contribute to cancer development and progression in various tissues.

## 7 Distinct functions of p85α and p85β on the immune system regulation

Evidence suggests divergent roles of p85 isoforms in immune function. Upon T cell activation through the T cell antigen receptor (TCR)/CD3 complex or protein kinase C (PKC), phosphorylation of p85β occurs in threonine residues. However, p85α remains unchanged during T cell activation (108). This initial finding led to subsequent studies highlighting the distinct functions of p85 isoforms in the immune system, with p85α in the function of B cells and p85β in the regulation of T cells.

Knockout of *pik3r1* in mice at the early stage leads to lethality, primarily attributed to defects in B cell proliferation (57, 109). These mice display downregulated expression of p110δ, the most prevalent form of p110 isoforms, in B cells (110). Consequently, the diminished p110δ levels lead to decreased proliferation, maturation and differentiation of B cells (111, 112). However, the absence of p85α does not appear to affect the development of T cells (57). In contrast to p85α, the deletion of p85β in mice does not impact the expression of p110α, p110β, and p110δ in B cells, nor does it affect B cell proliferation (112). However, p85α/β double knockout increases B cell proliferation compared to p85α knockout, which implies p85β acts to negatively regulate the role of p85α on B cells (113). Whole-body or B cell-specific deletion of p85α results in reduced phosphorylation of Akt at threonine 308 and serine 473 residues in B cells, compared to wild-type (110, 113). B cell-specific knockout of p85β results in comparable levels of increased Akt phosphorylation at serine 473 compared to wild-type (113). p85α/β double knockout further decreases Akt phosphorylation, compared to p85α knockout mice. These findings indicate that p85α is the primary isoform responsible for regulating Akt activity in B cells. However, p85β can play a role in modulating Akt activity when p85α is absent (113). Additionally, p85α is necessary for BCR-stimulated calcium mobilization, which is an essential process for cellular signaling and physiological functions. However, p85β is not essential for this response, even when p85α is absent (113). These demonstrate that p85α and p85β exert differential effects on specific signaling pathways in B cells.

Studies have shown that p85β is responsible for the regulation of T cells. CD28 is a T cell receptor that plays a role in T cell differentiation into long-term memory T cells. CD28-deficient T cells exhibit incomplete primary activation and impaired T cell differentiation. p85β has been found to exhibit a higher affinity for CD28, compared to p85α (114). It also downregulates the expression of casitas B-lineage lymphoma (CBL) ubiquitin ligases, which serve as a negative regulator of T-cell activation. In the absence of p85β, primary T cell activation does not lead to activation of PI3K, nor downregulate CBL proteins, such as c-CBL and CBL-b, resulting in impaired differentiation of activated T cells. Although their primary immune response to antigen was slightly enhanced, the secondary immune response in CD4^+^ spleen and lymph nodes was significantly decreased, suggesting the involvement of p85β in the secondary immune response (114). In activated p85β-deficient T cells induced by anti-CD3 and interleukin-2 (IL-2), the activity of caspase-6, a key protein that triggers apoptosis, was reduced, indicating the role of p85β in the cell death pathways in T cells (112). Additionally, enhanced proliferation was observed in these cells upon stimulation with anti-CD3 and IL-2, as shown by a higher number of cell divisions of CD4^+^ and CD8^+^ T cells (112). In conclusion, previous findings indicate the role of p85β in modulating the proliferation, maturation, and differentiation of T cells.

## 8 Discussion

Investigating the roles of p85 has provided valuable insights for various pathological conditions. Developing a more specific therapeutic strategy with known mechanisms can lead to even more effective remedies with fewer unexpected side effects arising from unknown biological processes. Genetic studies and approaches to target specific genes have greatly contributed to recent understanding. However, these approaches require careful evaluation, taking into account target tissues and metabolic parameters based on previous reports that show various consequences.

This review provides a comprehensive overview of the roles played by the regulatory subunits of class IA PI3K in metabolism, cancer, and the immune system. Despite their structural similarity, p85α and p85β demonstrate significant functional differences. While p85α is primarily responsive to insulin, p85β does not exhibit the same level of responsiveness (65, 83). p85α acts as a tumor suppressor, whereas p85β functions as an oncogene (97, 104). p85α influences B cell development, while p85β is involved in regulating T cells (112, 114). The cSH2 domain of p85α downregulates PI3K signaling, whereas the cSH2 domain of p85β upregulates PI3K signaling (115). Only p85β facilitates the nuclear translocation of p110β (116), an isoform of p110 that regulates DNA repairs (117), and oncogenic transformation (118). p85α splicing variants also exhibit different functions. Upregulation of p85α in liver-specific *pik3r1* knockout mice impairs glucose tolerance, while hepatic reconstitution of p50α or p55α in these knockout mice has no effect on glucose tolerance (67).

Protein function can vary significantly depending on specific cell types due to distinct molecular compositions, gene expression patterns, and physiological requirements. Understanding the diverse outcomes exhibited by a protein in different tissue types is crucial, underscoring the need to investigate its role across various tissues. For instance, the knockout of p85α in the liver improves insulin sensitivity (78), while its absence has no effect on insulin sensitivity in skeletal muscle (77). Mice with a deletion of p85α in the presence of p50α exhibit increased insulin sensitivity in muscles, but not in the liver (72). Furthermore, p85α levels are increased in the skeletal muscle of obese and type 2 diabetic individuals (119, 120) and in adipose tissues of high-fat diet-fed obese mice (65), but decreased in the liver of high-fat diet-induced obese and genetically obese *ob*/*ob* mice (83, 121), suggesting different effects of p85α in those tissues. This knowledge is vital for developing targeted therapeutic interventions and advancing our understanding of complex biological systems.

Animal studies that employ high-fat diets to induce metabolic diseases can reveal phenotypic and mechanistic differences compared to control diets. Studies in lean conditions provide insights into normal physiological processes and serve as a baseline for understanding the impact of genetic alterations. On the other hand, studies in obese conditions help elucidate the molecular and cellular changes associated with metabolic disorders and their potential therapeutic targets. They allow to understand how biological processes and protein functions are influenced by different metabolic states. Additionally, recent studies have highlighted the importance of considering fiber content in diets (122).

The field continues to require further in-depth investigations to advance our understanding and uncover additional insights into the complex functions of these regulatory subunits. Exploring the intricate mechanisms of PI3K signaling holds immense potential for therapeutic advancements.

## Author contributions

C-WK and SP conceptualized, wrote, and edited the manuscript, and JL contributed to the literature search and editing. All authors contributed to the article and approved the submitted version.

## References

[1] Engelman JA Luo J Cantley LC. The evolution of phosphatidylinositol 3-kinases as regulators of growth and metabolism. Nat Rev Genet (2006) 7(8):606–19. doi: 10.1038/nrg1879 16847462

[2] Chen L Janetopoulos C Huang YE Iijima M Borleis J Devreotes PN. Two phases of actin polymerization display different dependencies on PI(3,4,5)P3 accumulation and have unique roles during chemotaxis. Mol Biol Cell (2003) 14(12):5028–37. doi: 10.1091/mbc.e03-05-0339 PMC284804 14595116

[3] Huang YE Iijima M Parent CA Funamoto S Firtel RA Devreotes P. Receptor-mediated regulation of PI3Ks confines PI(3,4,5)P3 to the leading edge of chemotaxing cells. Mol Biol Cell (2003) 14(5):1913–22. doi: 10.1091/mbc.e02-10-0703 PMC165086 12802064

[4] Funamoto S Meili R Lee S Parry L Firtel RA. Spatial and temporal regulation of 3-phosphoinositides by PI 3-kinase and PTEN mediates chemotaxis. Cell (2002) 109(5):611–23. doi: 10.1016/S0092-8674(02)00755-9 12062104

[5] Hirsch E Katanaev VL Garlanda C Azzolino O Pirola L Silengo L. Central role for G protein-coupled phosphoinositide 3-kinase gamma in inflammation. Science (2000) 287(5455):1049–53. doi: 10.1126/science.287.5455.1049 10669418

[6] Cantley LC. The phosphoinositide 3-kinase pathway. Science (2002) 296(5573):1655–7. doi: 10.1126/science.296.5573.1655 12040186

[7] Leevers SJ Vanhaesebroeck B Waterfield MD. Signalling through phosphoinositide 3-kinases: the lipids take centre stage. Curr Opin Cell Biol (1999) 11(2):219–25. doi: 10.1016/S0955-0674(99)80029-5 10209156

[8] Zvelebil MJ MacDougall L Leevers S Volinia S Vanhaesebroeck B Gout I. Structural and functional diversity of phosphoinositide 3-kinases. Philos Trans R Soc Lond B Biol Sci (1996) 351(1336):217–23. doi: 10.1098/rstb.1996.0019 8650269

[9] Fruman DA Cantley LC Carpenter CL. Structural organization and alternative splicing of the murine phosphoinositide 3-kinase p85 alpha gene. Genomics (1996) 37(1):113–21. doi: 10.1006/geno.1996.0527 8921377

[10] Cortes I Sanchez-Ruiz J Zuluaga S Calvanese V Marques M Hernandez C. p85beta phosphoinositide 3-kinase subunit regulates tumor progression. Proc Natl Acad Sci USA (2012) 109(28):11318–23. doi: 10.1073/pnas.1118138109 PMC3396516 22733740

[11] Inukai K Anai M Van Breda E Hosaka T Katagiri H Funaki M. A novel 55-kDa regulatory subunit for phosphatidylinositol 3-kinase structurally similar to p55PIK Is generated by alternative splicing of the p85alpha gene. J Biol Chem (1996) 271(10):5317–20. doi: 10.1074/jbc.271.10.5317 8621382

[12] Suire S Coadwell J Ferguson GJ Davidson K Hawkins P Stephens L. p84, a new Gbetagamma-activated regulatory subunit of the type IB phosphoinositide 3-kinase p110gamma. Curr Biol (2005) 15(6):566–70. doi: 10.1016/j.cub.2005.02.020 15797027

[13] Voigt P Dorner MB Schaefer M. Characterization of p87PIKAP, a novel regulatory subunit of phosphoinositide 3-kinase gamma that is highly expressed in heart and interacts with PDE3B. J Biol Chem (2006) 281(15):9977–86. doi: 10.1074/jbc.M512502200 16476736

[14] Luo J Manning BD Cantley LC. Targeting the PI3K-Akt pathway in human cancer: rationale and promise. Cancer Cell (2003) 4(4):257–62. doi: 10.1016/S1535-6108(03)00248-4 14585353

[15] Huang W Jiang D Wang X Wang K Sims CE Allbritton NL. Kinetic analysis of PI3K reactions with fluorescent PIP2 derivatives. Anal Bioanal Chem (2011) 401(6):1881–8. doi: 10.1007/s00216-011-5257-z PMC3311999 21789487

[16] Carpenter CL Duckworth BC Auger KR Cohen B Schaffhausen BS Cantley LC. Purification and characterization of phosphoinositide 3-kinase from rat liver. J Biol Chem (1990) 265(32):19704–11. doi: 10.1016/S0021-9258(17)45429-9 2174051

[17] Katso R Okkenhaug K Ahmadi K White S Timms J Waterfield MD. Cellular function of phosphoinositide 3-kinases: implications for development, homeostasis, and cancer. Annu Rev Cell Dev Biol (2001) 17:615–75. doi: 10.1146/annurev.cellbio.17.1.615 11687500

[18] Jean S Kiger AA. Classes of phosphoinositide 3-kinases at a glance. J Cell Sci (2014) 127(Pt 5):923–8. doi: 10.1242/jcs.093773 PMC3937771 24587488

[19] Vanhaesebroeck B Leevers SJ Panayotou G Waterfield MD. Phosphoinositide 3-kinases: a conserved family of signal transducers. Trends Biochem Sci (1997) 22(7):267–72. doi: 10.1016/S0968-0004(97)01061-X 9255069

[20] Domin J Gaidarov I Smith ME Keen JH Waterfield MD. The class II phosphoinositide 3-kinase PI3K-C2alpha is concentrated in the trans-Golgi network and present in clathrin-coated vesicles. J Biol Chem (2000) 275(16):11943–50. doi: 10.1074/jbc.275.16.11943 10766823

[21] Gozzelino L De Santis MC Gulluni F Hirsch E Martini M. PI(3,4)P2 signaling in cancer and metabolism. Front Oncol (2020) 10:360. doi: 10.3389/fonc.2020.00360 32296634 PMC7136497

[22] Backer JM. The regulation and function of Class III PI3Ks: novel roles for Vps34. Biochem J (2008) 410(1):1–17. doi: 10.1042/BJ20071427 18215151

[23] Yan Y Flinn RJ Wu H Schnur RS Backer JM. hVps15, but not Ca2+/CaM, is required for the activity and regulation of hVps34 in mammalian cells. Biochem J (2009) 417(3):747–55. doi: 10.1042/BJ20081865 PMC2652830 18957027

[24] Stack JH Emr SD. Vps34p required for yeast vacuolar protein sorting is a multiple specificity kinase that exhibits both protein kinase and phosphatidylinositol-specific PI 3-kinase activities. J Biol Chem (1994) 269(50):31552–62. doi: 10.1016/S0021-9258(18)31729-0 7989323

[25] Sugimoto Y Whitman M Cantley LC Erikson RL. Evidence that the Rous sarcoma virus transforming gene product phosphorylates phosphatidylinositol and diacylglycerol. Proc Natl Acad Sci USA (1984) 81(7):2117–21. doi: 10.1073/pnas.81.7.2117 PMC345448 6326105

[26] Yang J Nie J Ma X Wei Y Peng Y Wei X. Targeting PI3K in cancer: mechanisms and advances in clinical trials. Mol Cancer (2019) 18(1):26. doi: 10.1186/s12943-019-0954-x 30782187 PMC6379961

[27] Calautti E Li J Saoncella S Brissette JL Goetinck PF. Phosphoinositide 3-kinase signaling to Akt promotes keratinocyte differentiation versus death. J Biol Chem (2005) 280(38):32856–65. doi: 10.1074/jbc.M506119200 16036919

[28] Franke TF Hornik CP Segev L Shostak GA Sugimoto C. PI3K/Akt and apoptosis: size matters. Oncogene (2003) 22(56):8983–98. doi: 10.1038/sj.onc.1207115 14663477

[29] Escobedo JA Navankasattusas S Kavanaugh WM Milfay D Fried VA Williams LT. cDNA cloning of a novel 85 kd protein that has SH2 domains and regulates binding of PI3-kinase to the PDGF beta-receptor. Cell (1991) 65(1):75–82. doi: 10.1016/0092-8674(91)90409-R 1849460

[30] Burke JE. Structural basis for regulation of phosphoinositide kinases and their involvement in human disease. Mol Cell (2018) 71(5):653–73. doi: 10.1016/j.molcel.2018.08.005 30193094

[31] Yu J Zhang Y McIlroy J Rordorf-Nikolic T Orr GA Backer JM. Regulation of the p85/p110 phosphatidylinositol 3’-kinase: stabilization and inhibition of the p110alpha catalytic subunit by the p85 regulatory subunit. Mol Cell Biol (1998) 18(3):1379–87. doi: 10.1128/MCB.18.3.1379 PMC108851 9488453

[32] Zhang X Vadas O Perisic O Anderson KE Clark J Hawkins PT. Structure of lipid kinase p110beta/p85beta elucidates an unusual SH2-domain-mediated inhibitory mechanism. Mol Cell (2011) 41(5):567–78. doi: 10.1016/j.molcel.2011.01.026 PMC3670040 21362552

[33] Burke JE Vadas O Berndt A Finegan T Perisic O Williams RL. Dynamics of the phosphoinositide 3-kinase p110delta interaction with p85alpha and membranes reveals aspects of regulation distinct from p110alpha. Structure (2011) 19(8):1127–37. doi: 10.1016/j.str.2011.06.003 PMC3155019 21827948

[34] Yu J Wjasow C Backer JM. Regulation of the p85/p110alpha phosphatidylinositol 3’-kinase. Distinct roles for the n-terminal and c-terminal SH2 domains. J Biol Chem (1998) 273(46):30199–203. doi: 10.1074/jbc.273.46.30199 9804776

[35] Backer JM Myers MG Jr. Shoelson SE Chin DJ Sun XJ Miralpeix M. Phosphatidylinositol 3’-kinase is activated by association with IRS-1 during insulin stimulation. EMBO J (1992) 11(9):3469–79. doi: 10.1002/j.1460-2075.1992.tb05426.x PMC556882 1380456

[36] Rordorf-Nikolic T Van Horn DJ Chen D White MF Backer JM. Regulation of phosphatidylinositol 3’-kinase by tyrosyl phosphoproteins. Full activation requires occupancy of both SH2 domains in the 85-kDa regulatory subunit. J Biol Chem (1995) 270(8):3662–6. doi: 10.1074/jbc.270.8.3662 7876105

[37] Cuevas BD Lu Y Mao M Zhang J LaPushin R Siminovitch K. Tyrosine phosphorylation of p85 relieves its inhibitory activity on phosphatidylinositol 3-kinase. J Biol Chem (2001) 276(29):27455–61. doi: 10.1074/jbc.M100556200 11337495

[38] Carlson CJ White MF Rondinone CM. Mammalian target of rapamycin regulates IRS-1 serine 307 phosphorylation. Biochem Biophys Res Commun (2004) 316(2):533–9. doi: 10.1016/j.bbrc.2004.02.082 15020250

[39] Gual P Le Marchand-Brustel Y Tanti JF. Positive and negative regulation of insulin signaling through IRS-1 phosphorylation. Biochimie (2005) 87(1):99–109. doi: 10.1016/j.biochi.2004.10.019 15733744

[40] Yu Y Yoon SO Poulogiannis G Yang Q Ma XM Villen J. Phosphoproteomic analysis identifies Grb10 as an mTORC1 substrate that negatively regulates insulin signaling. Science (2011) 332(6035):1322–6. doi: 10.1126/science.1199484 PMC3195509 21659605

[41] Wang L Balas B Christ-Roberts CY Kim RY Ramos FJ Kikani CK. Peripheral disruption of the Grb10 gene enhances insulin signaling and sensitivity in vivo. Mol Cell Biol (2007) 27(18):6497–505. doi: 10.1128/MCB.00679-07 PMC2099625 17620412

[42] Comb WC Hutti JE Cogswell P Cantley LC Baldwin AS. p85alpha SH2 domain phosphorylation by IKK promotes feedback inhibition of PI3K and Akt in response to cellular starvation. Mol Cell (2012) 45(6):719–30. doi: 10.1016/j.molcel.2012.01.010 PMC3319231 22342344

[43] Kearney AL Norris DM Ghomlaghi M Kin Lok Wong M Humphrey SJ Carroll L. Akt phosphorylates insulin receptor substrate to limit PI3K-mediated PIP3 synthesis. Elife (2021) 10:e66942. doi: 10.7554/eLife.66942 34253290 PMC8277355

[44] O’Reilly KE Rojo F She QB Solit D Mills GB Smith D. mTOR inhibition induces upstream receptor tyrosine kinase signaling and activates Akt. Cancer Res (2006) 66(3):1500–8. doi: 10.1158/0008-5472.CAN-05-2925 PMC3193604 16452206

[45] Chandarlapaty S Sawai A Scaltriti M Rodrik-Outmezguine V Grbovic-Huezo O Serra V. AKT inhibition relieves feedback suppression of receptor tyrosine kinase expression and activity. Cancer Cell (2011) 19(1):58–71. doi: 10.1016/j.ccr.2010.10.031 21215704 PMC3025058

[46] Chakrabarty A Sanchez V Kuba MG Rinehart C Arteaga CL. Feedback upregulation of HER3 (ErbB3) expression and activity attenuates antitumor effect of PI3K inhibitors. Proc Natl Acad Sci USA (2012) 109(8):2718–23. doi: 10.1073/pnas.1018001108 PMC3286932 21368164

[47] Mukherjee R Vanaja KG Boyer JA Gadal S Solomon H Chandarlapaty S. Regulation of PTEN translation by PI3K signaling maintains pathway homeostasis. Mol Cell (2021) 81(4):708–23 e5. doi: 10.1016/j.molcel.2021.01.033 33606974 PMC8384339

[48] White MF Kahn CR. Insulin action at a molecular level - 100 years of progress. Mol Metab (2021) 52:101304. doi: 10.1016/j.molmet.2021.101304 34274528 PMC8551477

[49] Georgescu MM. PTEN tumor suppressor network in PI3K-akt pathway control. Genes Cancer (2010) 1(12):1170–7. doi: 10.1177/1947601911407325 PMC3092286 21779440

[50] Dimmeler S Fleming I Fisslthaler B Hermann C Busse R Zeiher AM. Activation of nitric oxide synthase in endothelial cells by Akt-dependent phosphorylation. Nature (1999) 399(6736):601–5. doi: 10.1038/21224 10376603

[51] Abid MR Guo S Minami T Spokes KC Ueki K Skurk C. Vascular endothelial growth factor activates PI3K/Akt/forkhead signaling in endothelial cells. Arterioscler Thromb Vasc Biol (2004) 24(2):294–300. doi: 10.1161/01.ATV.0000110502.10593.06 14656735

[52] Papapetropoulos A Garcia-Cardena G Madri JA Sessa WC. Nitric oxide production contributes to the angiogenic properties of vascular endothelial growth factor in human endothelial cells. J Clin Invest (1997) 100(12):3131–9. doi: 10.1172/JCI119868 PMC508526 9399960

[53] Li Z Yang Z Passaniti A Lapidus RG Liu X Cullen KJ. A positive feedback loop involving EGFR/Akt/mTORC1 and IKK/NF-kB regulates head and neck squamous cell carcinoma proliferation. Oncotarget (2016) 7(22):31892–906. doi: 10.18632/oncotarget.7441 PMC5077984 26895469

[54] Fracchiolla D Chang C Hurley JH. Martens S. A PI3K-WIPI2 positive feedback loop allosterically activates LC3 lipidation in autophagy. J Cell Biol (2020) 219(7):e201912098. doi: 10.1083/jcb.201912098 32437499 PMC7337497

[55] Panchamoorthy G Fukazawa T Miyake S Soltoff S Reedquist K Druker B. p120cbl is a major substrate of tyrosine phosphorylation upon B cell antigen receptor stimulation and interacts in *vivo* with Fyn and Syk tyrosine kinases, Grb2 and Shc adaptors, and the p85 subunit of phosphatidylinositol 3-kinase. J Biol Chem (1996) 271(6):3187–94. doi: 10.1074/jbc.271.6.3187 8621719

[56] Luo J Cantley LC. The negative regulation of phosphoinositide 3-kinase signaling by p85 and it’s implication in cancer. Cell Cycle (2005) 4(10):1309–12. doi: 10.4161/cc.4.10.2062 16131837

[57] Fruman DA Snapper SB Yballe CM Davidson L Yu JY Alt FW. Impaired B cell development and proliferation in absence of phosphoinositide 3-kinase p85alpha. Science (1999) 283(5400):393–7. doi: 10.1126/science.283.5400.393 9888855

[58] Giorgetti S Ballotti R Kowalski-Chauvel A Cormont M Van Obberghen E. Insulin stimulates phosphatidylinositol-3-kinase activity in rat adipocytes. Eur J Biochem (1992) 207(2):599–606. doi: 10.1111/j.1432-1033.1992.tb17086.x 1321717

[59] Otsu M Hiles I Gout I Fry MJ Ruiz-Larrea F Panayotou G. Characterization of two 85 kd proteins that associate with receptor tyrosine kinases, middle-T/pp60c-src complexes, and PI3-kinase. Cell (1991) 65(1):91–104. doi: 10.1016/0092-8674(91)90411-Q 1707345

[60] Fruman DA Mauvais-Jarvis F Pollard DA Yballe CM Brazil D Bronson RT. Hypoglycaemia, liver necrosis and perinatal death in mice lacking all isoforms of phosphoinositide 3-kinase p85 alpha. Nat Genet (2000) 26(3):379–82. doi: 10.1038/81715 11062485

[61] Terauchi Y Tsuji Y Satoh S Minoura H Murakami K Okuno A. Increased insulin sensitivity and hypoglycaemia in mice lacking the p85 alpha subunit of phosphoinositide 3-kinase. Nat Genet (1999) 21(2):230–5. doi: 10.1038/6023 9988280

[62] Barbour LA Mizanoor Rahman S Gurevich I Leitner JW Fischer SJ Roper MD. Increased P85alpha is a potent negative regulator of skeletal muscle insulin signaling and induces in *vivo* insulin resistance associated with growth hormone excess. J Biol Chem (2005) 280(45):37489–94. doi: 10.1074/jbc.M506967200 16166093

[63] Terauchi Y Matsui J Kamon J Yamauchi T Kubota N Komeda K. Increased serum leptin protects from adiposity despite the increased glucose uptake in white adipose tissue in mice lacking p85alpha phosphoinositide 3-kinase. Diabetes (2004) 53(9):2261–70. doi: 10.2337/diabetes.53.9.2261 15331535

[64] Mauvais-Jarvis F Ueki K Fruman DA Hirshman MF Sakamoto K Goodyear LJ. Reduced expression of the murine p85alpha subunit of phosphoinositide 3-kinase improves insulin signaling and ameliorates diabetes. J Clin Invest (2002) 109(1):141–9. doi: 10.1172/JCI0213305 PMC150818 11781359

[65] McCurdy CE Schenk S Holliday MJ Philp A Houck JA Patsouris D. Attenuated Pik3r1 expression prevents insulin resistance and adipose tissue macrophage accumulation in diet-induced obese mice. Diabetes (2012) 61(10):2495–505. doi: 10.2337/db11-1433 PMC3447911 22698915

[66] Taniguchi CM Tran TT Kondo T Luo J Ueki K Cantley LC. Phosphoinositide 3-kinase regulatory subunit p85alpha suppresses insulin action via positive regulation of PTEN. Proc Natl Acad Sci USA (2006) 103(32):12093–7. doi: 10.1073/pnas.0604628103 PMC1524929 16880400

[67] Taniguchi CM Aleman JO Ueki K Luo J Asano T Kaneto H. The p85alpha regulatory subunit of phosphoinositide 3-kinase potentiates c-Jun N-terminal kinase-mediated insulin resistance. Mol Cell Biol (2007) 27(8):2830–40. doi: 10.1128/MCB.00079-07 PMC1899914 17283057

[68] Gomez-Hernandez A Lopez-Pastor AR Rubio-Longas C Majewski P Beneit N Viana-Huete V. Specific knockout of p85alpha in brown adipose tissue induces resistance to high-fat diet-induced obesity and its metabolic complications in male mice. Mol Metab (2020) 31:1–13. doi: 10.1016/j.molmet.2019.10.010 31918912 PMC6977168

[69] Inukai K Funaki M Anai M Ogihara T Katagiri H Fukushima Y. Five isoforms of the phosphatidylinositol 3-kinase regulatory subunit exhibit different associations with receptor tyrosine kinases and their tyrosine phosphorylations. FEBS Lett (2001) 490(1-2):32–8. doi: 10.1016/S0014-5793(01)02132-9 11172806

[70] Inukai K Funaki M Ogihara T Katagiri H Kanda A Anai M. p85alpha gene generates three isoforms of regulatory subunit for phosphatidylinositol 3-kinase (PI 3-Kinase), p50alpha, p55alpha, and p85alpha, with different PI 3-kinase activity elevating responses to insulin. J Biol Chem (1997) 272(12):7873–82. doi: 10.1074/jbc.272.12.7873 9065454

[71] Ueki K Algenstaedt P Mauvais-Jarvis F Kahn CR. Positive and negative regulation of phosphoinositide 3-kinase-dependent signaling pathways by three different gene products of the p85alpha regulatory subunit. Mol Cell Biol (2000) 20(21):8035–46. doi: 10.1128/MCB.20.21.8035-8046.2000 PMC86414 11027274

[72] Aoki K Matsui J Kubota N Nakajima H Iwamoto K Takamoto I. Role of the liver in glucose homeostasis in PI 3-kinase p85alpha-deficient mice. Am J Physiol Endocrinol Metab (2009) 296(4):E842–53. doi: 10.1152/ajpendo.90528.2008 19176357

[73] Chen D Mauvais-Jarvis F Bluher M Fisher SJ Jozsi A Goodyear LJ. p50alpha/p55alpha phosphoinositide 3-kinase knockout mice exhibit enhanced insulin sensitivity. Mol Cell Biol (2004) 24(1):320–9. doi: 10.1128/MCB.24.1.320-329.2004 PMC303335 14673165

[74] Bergen HT Monkman N Mobbs CV. Injection with gold thioglucose impairs sensitivity to glucose: evidence that glucose-responsive neurons are important for long-term regulation of body weight. Brain Res (1996) 734(1-2):332–6. doi: 10.1016/0006-8993(96)00887-6 8896843

[75] Heydrick SJ Gautier N Olichon-Berthe C Van Obberghen E Le Marchand-Brustel Y. Early alteration of insulin stimulation of PI 3-kinase in muscle and adipocyte from gold thioglucose obese mice. Am J Physiol (1995) 268(4 Pt 1):E604–12. doi: 10.1152/ajpendo.1995.268.4.E604 7733258

[76] Martins VF Tahvilian S Kang JH Svensson K Hetrick B Chick WS. Calorie restriction-induced increase in skeletal muscle insulin sensitivity is not prevented by overexpression of the p55alpha subunit of phosphoinositide 3-kinase. Front Physiol (2018) 9:789. doi: 10.3389/fphys.2018.00789 29997524 PMC6030672

[77] Luo J Sobkiw CL Hirshman MF Logsdon MN Li TQ Goodyear LJ. Loss of class IA PI3K signaling in muscle leads to impaired muscle growth, insulin response, and hyperlipidemia. Cell Metab (2006) 3(5):355–66. doi: 10.1016/j.cmet.2006.04.003 16679293

[78] Park SW Zhou Y Lee J Lu A Sun C Chung J. The regulatory subunits of PI3K, p85alpha and p85beta, interact with XBP-1 and increase its nuclear translocation. Nat Med (2010) 16(4):429–37. doi: 10.1038/nm.2099 PMC3071012 20348926

[79] Ueki K Yballe CM Brachmann SM Vicent D Watt JM Kahn CR. Increased insulin sensitivity in mice lacking p85beta subunit of phosphoinositide 3-kinase. Proc Natl Acad Sci USA (2002) 99(1):419–24. doi: 10.1073/pnas.012581799 PMC117575 11752399

[80] Taniguchi CM Kondo T Sajan M Luo J Bronson R Asano T. Divergent regulation of hepatic glucose and lipid metabolism by phosphoinositide 3-kinase via Akt and PKClambda/zeta. Cell Metab (2006) 3(5):343–53. doi: 10.1016/j.cmet.2006.04.005 16679292

[81] Cuppen E van Ham M Pepers B Wieringa B Hendriks W. Identification and molecular characterization of BP75, a novel bromodomain-containing protein. FEBS Lett (1999) 459(3):291–8. doi: 10.1016/S0014-5793(99)01191-6 10526152

[82] Park SW Herrema H Salazar M Cakir I Cabi S Basibuyuk Sahin F. BRD7 regulates XBP1s’ activity and glucose homeostasis through its interaction with the regulatory subunits of PI3K. Cell Metab (2014) 20(1):73–84. doi: 10.1016/j.cmet.2014.04.006 24836559 PMC4079724

[83] Lee JM Liu R Park SW. The regulatory subunits of PI3K, p85alpha and p85beta, differentially affect BRD7-mediated regulation of insulin signaling. J Mol Cell Biol (2022) 13(12):889–901. doi: 10.1093/jmcb/mjab073 34751372 PMC8800525

[84] Chiefari E Foti DP Sgarra R Pegoraro S Arcidiacono B Brunetti FS. Transcriptional regulation of glucose metabolism: the emerging role of the HMGA1 chromatin factor. Front Endocrinol (Lausanne) (2018) 9:357. doi: 10.3389/fendo.2018.00357 30034366 PMC6043803

[85] Brachmann SM Ueki K Engelman JA Kahn RC Cantley LC. Phosphoinositide 3-kinase catalytic subunit deletion and regulatory subunit deletion have opposite effects on insulin sensitivity in mice. Mol Cell Biol (2005) 25(5):1596–607. doi: 10.1128/MCB.25.5.1596-1607.2005 PMC549361 15713620

[86] Cheung LW Walkiewicz KW Besong TM Guo H Hawke DH Arold ST. Regulation of the PI3K pathway through a p85alpha monomer-homodimer equilibrium. Elife (2015) 4:e06866. doi: 10.7554/eLife.06866 26222500 PMC4518712

[87] LoPiccolo J Kim SJ Shi Y Wu B Wu H Chait BT. Assembly and molecular architecture of the phosphoinositide 3-kinase p85alpha homodimer. J Biol Chem (2015) 290(51):30390–405. doi: 10.1074/jbc.M115.689604 PMC4683262 26475863

[88] de la Cruz-Herrera CF Baz-Martinez M Lang V El Motiam A Barbazan J Couceiro R. Conjugation of SUMO to p85 leads to a novel mechanism of PI3K regulation. Oncogene (2016) 35(22):2873–80. doi: 10.1038/onc.2015.356 26411363

[89] Hayashi H Nishioka Y Kamohara S Kanai F Ishii K Fukui Y. The alpha-type 85-kDa subunit of phosphatidylinositol 3-kinase is phosphorylated at tyrosines 368, 580, and 607 by the insulin receptor. J Biol Chem (1993) 268(10):7107–17. doi: 10.1016/S0021-9258(18)53152-5 8385099

[90] Clayton NS Fox M Vicente-Garcia JJ Schroeder CM Littlewood TD Wilde JI. Assembly of nuclear dimers of PI3K regulatory subunits is regulated by the Cdc42-activated tyrosine kinase ACK. J Biol Chem (2022) 298(6):101916. doi: 10.1016/j.jbc.2022.101916 35429500 PMC9127371

[91] Ueki K Fruman DA Brachmann SM Tseng YH Cantley LC Kahn CR. Molecular balance between the regulatory and catalytic subunits of phosphoinositide 3-kinase regulates cell signaling and survival. Mol Cell Biol (2002) 22(3):965–77. doi: 10.1128/MCB.22.3.965-977.2002 PMC133541 11784871

[92] Tsolakos N Durrant TN Chessa T Suire SM Oxley D Kulkarni S. Quantitation of class IA PI3Ks in mice reveals p110-free-p85s and isoform-selective subunit associations and recruitment to receptors. Proc Natl Acad Sci USA (2018) 115(48):12176–81. doi: 10.1073/pnas.1803446115 PMC6275495 30442661

[93] Luo J Field SJ Lee JY Engelman JA Cantley LC. The p85 regulatory subunit of phosphoinositide 3-kinase down-regulates IRS-1 signaling via the formation of a sequestration complex. J Cell Biol (2005) 170(3):455–64. doi: 10.1083/jcb.200503088 PMC2171479 16043515

[94] Chiu YH Lee JY Cantley LC. BRD7, a tumor suppressor, interacts with p85alpha and regulates PI3K activity. Mol Cell (2014) 54(1):193–202. doi: 10.1016/j.molcel.2014.02.016 24657164 PMC4004185

[95] Park SW Lee JM. Emerging roles of BRD7 in pathophysiology. Int J Mol Sci (2020) 21(19):7127. doi: 10.3390/ijms21197127 32992509 PMC7583729

[96] Winnay JN Boucher J Mori MA Ueki K Kahn CR. A regulatory subunit of phosphoinositide 3-kinase increases the nuclear accumulation of X-box-binding protein-1 to modulate the unfolded protein response. Nat Med (2010) 16(4):438–45. doi: 10.1038/nm.2121 PMC4371606 20348923

[97] Taniguchi CM Winnay J Kondo T Bronson RT Guimaraes AR Aleman JO. The phosphoinositide 3-kinase regulatory subunit p85alpha can exert tumor suppressor properties through negative regulation of growth factor signaling. Cancer Res (2010) 70(13):5305–15. doi: 10.1158/0008-5472.CAN-09-3399 PMC3204358 20530665

[98] Chen Y Zeng C Zhan Y Wang H Jiang X Li W. Aberrant low expression of p85alpha in stromal fibroblasts promotes breast cancer cell metastasis through exosome-mediated paracrine Wnt10b. Oncogene (2017) 36(33):4692–705. doi: 10.1038/onc.2017.100 PMC5562851 28394344

[99] Thorpe LM Spangle JM Ohlson CE Cheng H Roberts TM Cantley LC. PI3K-p110alpha mediates the oncogenic activity induced by loss of the novel tumor suppressor PI3K-p85alpha. Proc Natl Acad Sci USA (2017) 114(27):7095–100. doi: 10.1073/pnas.1704706114 PMC5502636 28630349

[100] Wang J Zhang N Peng M Hua X Huang C Tian Z. p85alpha inactivates MMP-2 and suppresses bladder cancer invasion by inhibiting MMP-14 transcription and TIMP-2 degradation. Neoplasia (2019) 21(9):908–20. doi: 10.1016/j.neo.2019.07.007 PMC6700442 31401412

[101] Jaiswal BS Janakiraman V Kljavin NM Chaudhuri S Stern HM Wang W. Somatic mutations in p85alpha promote tumorigenesis through class IA PI3K activation. Cancer Cell (2009) 16(6):463–74. doi: 10.1016/j.ccr.2009.10.016 PMC2804903 19962665

[102] Vallejo-Diaz J Olazabal-Moran M Cariaga-Martinez AE Pajares MJ Flores JM Pio R. Targeted depletion of PIK3R2 induces regression of lung squamous cell carcinoma. Oncotarget (2016) 7(51):85063–78. doi: 10.18632/oncotarget.13195 PMC5356720 27835880

[103] Rao L Mak VCY Zhou Y Zhang D Li X Fung CCY. p85beta regulates autophagic degradation of AXL to activate oncogenic signaling. Nat Commun (2020) 11(1):2291. doi: 10.1038/s41467-020-16061-7 32385243 PMC7210311

[104] Ito Y Hart JR Ueno L Vogt PK. Oncogenic activity of the regulatory subunit p85beta of phosphatidylinositol 3-kinase (PI3K). Proc Natl Acad Sci USA (2014) 111(47):16826–9. doi: 10.1073/pnas.1420281111 PMC4250105 25385636

[105] Zhu N Zhang D Xie H Zhou Z Chen H Hu T. Endothelial-specific intron-derived miR-126 is down-regulated in human breast cancer and targets both VEGFA and PIK3R2. Mol Cell Biochem (2011) 351(1-2):157–64. doi: 10.1007/s11010-011-0723-7 21249429

[106] Cheung LW Hennessy BT Li J Yu S Myers AP Djordjevic B. High frequency of PIK3R1 and PIK3R2 mutations in endometrial cancer elucidates a novel mechanism for regulation of PTEN protein stability. Cancer Discovery (2011) 1(2):170–85. doi: 10.1158/2159-8290.CD-11-0039 PMC3187555 21984976

[107] Hao Y He B Wu L Li Y Wang C Wang T. Nuclear translocation of p85beta promotes tumorigenesis of PIK3CA helical domain mutant cancer. Nat Commun (2022) 13(1):1974. doi: 10.1038/s41467-022-29585-x 35418124 PMC9007954

[108] Reif K Gout I Waterfield MD Cantrell DA. Divergent regulation of phosphatidylinositol 3-kinase P85 alpha and P85 beta isoforms upon T cell activation. J Biol Chem (1993) 268(15):10780–8. doi: 10.1016/S0021-9258(18)82053-1 8388374

[109] Suzuki H Terauchi Y Fujiwara M Aizawa S Yazaki Y Kadowaki T. Xid-like immunodeficiency in mice with disruption of the p85alpha subunit of phosphoinositide 3-kinase. Science (1999) 283(5400):390–2. doi: 10.1126/science.283.5400.390 9888854

[110] Suzuki H Matsuda S Terauchi Y Fujiwara M Ohteki T Asano T. PI3K and Btk differentially regulate B cell antigen receptor-mediated signal transduction. Nat Immunol (2003) 4(3):280–6. doi: 10.1038/ni890 12563258

[111] Jou ST Carpino N Takahashi Y Piekorz R Chao JR Carpino N. Essential, nonredundant role for the phosphoinositide 3-kinase p110delta in signaling by the B-cell receptor complex. Mol Cell Biol (2002) 22(24):8580–91. doi: 10.1128/MCB.22.24.8580-8591.2002 PMC139888 12446777

[112] Deane JA Trifilo MJ Yballe CM Choi S Lane TE Fruman DA. Enhanced T cell proliferation in mice lacking the p85beta subunit of phosphoinositide 3-kinase. J Immunol (2004) 172(11):6615–25. doi: 10.4049/jimmunol.172.11.6615 15153476

[113] Oak JS Chen J Peralta RQ Deane JA Fruman DA. The p85beta regulatory subunit of phosphoinositide 3-kinase has unique and redundant functions in B cells. Autoimmunity (2009) 42(5):447–58. doi: 10.1080/08916930902911746 PMC2804088 19811262

[114] Alcazar I Cortes I Zaballos A Hernandez C Fruman DA Barber DF. p85beta phosphoinositide 3-kinase regulates CD28 coreceptor function. Blood (2009) 113(14):3198–208. doi: 10.1182/blood-2008-04-152942 PMC2945823 19190244

[115] Ito Y Vogt PK Hart JR. Domain analysis reveals striking functional differences between the regulatory subunits of phosphatidylinositol 3-kinase (PI3K), p85alpha and p85beta. Oncotarget (2017) 8(34):55863–76. doi: 10.18632/oncotarget.19866 PMC5593529 28915558

[116] Kumar A Redondo-Munoz J Perez-Garcia V Cortes I Chagoyen M Carrera AC. Nuclear but not cytosolic phosphoinositide 3-kinase beta has an essential function in cell survival. Mol Cell Biol (2011) 31(10):2122–33. doi: 10.1128/MCB.01313-10 PMC3133359 21383062

[117] Kumar A Fernandez-Capetillo O Carrera AC. Nuclear phosphoinositide 3-kinase beta controls double-strand break DNA repair. Proc Natl Acad Sci U S A (2010) 107(16):7491–6. doi: 10.1073/pnas.0914242107 PMC2867755 20368419

[118] Kang S Denley A Vanhaesebroeck B Vogt PK. Oncogenic transformation induced by the p110beta, -gamma, and -delta isoforms of class I phosphoinositide 3-kinase. Proc Natl Acad Sci USA (2006) 103(5):1289–94. doi: 10.1073/pnas.0510772103 PMC1360601 16432180

[119] Friedman JE Ishizuka T Shao J Huston L Highman T Catalano P. Impaired glucose transport and insulin receptor tyrosine phosphorylation in skeletal muscle from obese women with gestational diabetes. Diabetes (1999) 48(9):1807–14. doi: 10.2337/diabetes.48.9.1807 10480612

[120] Bandyopadhyay GK Yu JG Ofrecio J Olefsky JM. Increased p85/55/50 expression and decreased phosphotidylinositol 3-kinase activity in insulin-resistant human skeletal muscle. Diabetes (2005) 54(8):2351–9. doi: 10.2337/diabetes.54.8.2351 16046301

[121] Kerouz NJ Horsch D Pons S Kahn CR. Differential regulation of insulin receptor substrates-1 and -2 (IRS-1 and IRS-2) and phosphatidylinositol 3-kinase isoforms in liver and muscle of the obese diabetic (ob/ob) mouse. J Clin Invest (1997) 100(12):3164–72. doi: 10.1172/JCI119872 PMC508530 9399964

[122] Pellizzon MA Ricci MR. The common use of improper control diets in diet-induced metabolic disease research confounds data interpretation: the fiber factor. Nutr Metab (Lond) (2018) 15:3. doi: 10.1186/s12986-018-0243-5 29371873 PMC5769545

